# The Effect of Latitudinal Variation on Shrimp Reproductive Strategies

**DOI:** 10.1371/journal.pone.0155266

**Published:** 2016-05-09

**Authors:** Madelon van de Kerk, Chanda Jones Littles, Omar Saucedo, Kai Lorenzen

**Affiliations:** 1 Department of Wildlife Ecology and Conservation, University of Florida, 110 Newins-Ziegler Hall, Gainesville, FL, 32611–0430, United States of America; 2 School of Natural Resources and Environment, University of Florida, 103 Black Hall, Gainesville, FL, 32611–0430, United States of America; 3 Department of Mathematics, University of Florida, 358 Little Hall, Gainesville, FL, 32611–0430, United States of America; 4 School of Forest Resources and Conservation, University of Florida, 7922 NW 71st Street, Gainesville, FL, 32653–9720, United States of America; Zhejiang University, CHINA

## Abstract

Reproductive strategies comprise the timing and frequency of reproductive events and the number of offspring per reproductive event, depending on factors such as climate conditions. Therefore, species that exhibit plasticity in the allocation of reproductive effort can alter their behavior in response to climate change. Studying how the reproductive strategy of species varies along the latitudinal gradient can help us understand and predict how they will respond to climate change. We investigated the effects of the temporal allocation of reproductive effort on the population size of brown shrimp (*Farfantepenaeus aztecus*) along a latitudinal gradient. Multiple shrimp species exhibit variation in their reproductive strategies, and given the economic importance of brown shrimp to the commercial fishing sector of the Unites States, changes in the timing of their reproduction could have significant economic and social consequences. We used a stage-based, density-dependent matrix population model tailored to the life history of brown shrimp. Shrimp growth rates and environmental carrying capacity were varied based on the seasonal climate conditions at different latitudes, and we estimated the population size at equilibrium. The length of the growing season increased with decreasing latitude and the reproductive strategy leading to the highest population size changed from one annual birth pulse with high reproductive output to continuous low-output reproduction. Hence, our model confirms the classical paradigm of continuous reproduction at low latitudes, with increased seasonality of the breeding period towards the poles. Our results also demonstrate the potential for variation in climate to affect the optimal reproductive strategy for achieving maximum population sizes. Certainly, understanding these dynamics may inform more comprehensive management strategies for commercially important species like brown shrimp.

## Introduction

Environmental heterogeneity, such as temporal and spatial variation in climate and weather patterns, can affect the optimal timing of reproductive effort [1–3], resulting in a wide variety of reproductive strategies among different species. Birth-flow populations reproduce year-round and occur mainly in areas where seasonal differences are small or have little effect on species survival rates [4]. Other species reproduce seasonally, so that all births occur within relatively short time periods, which we call birth-pulses or reproductive peaks. These peaks often occur right before or early in the season that is most favorable for offspring survival, to optimize the reproductive output. Although most birth-pulse populations have one reproductive peak per year, there are also species that reproduce twice a year, or biannually [5–7].

Many species exhibit plasticity in how they temporally allocate their reproductive effort to adapt to seasonal changes in environmental factors such as temperature, precipitation, and food availability [8]. Therefore, species with ranges that include different climate zones may exhibit different reproductive strategies in different parts of their range [1,9,10]. When the optimal reproductive strategy varies along a species’ climatic range, their reproductive strategy may vary along a latitudinal gradient [4,10]. Studying how reproductive strategies vary along an animal’s latitudinal range may allow us to better understand potential adaptations to projected climate change.

Brown shrimp (*Farfantepenaeus aztecus*) are in the Penaeid shrimp family and occur over a fairly wide range of latitudes, along the Atlantic coast of the USA from Massachusetts down to the Florida Keys, and along the Gulf coast down to Yucatan, Mexico. Together with the two other species of Penaeid shrimp, the pink (*Farfantepenaeus duorarum*) and white shrimp (*Litopenaeus setiferus*), they make up over 90% of the shrimp harvested in the southeastern United States, where the commercial shrimp fishery is one of the most economically important fisheries. Like many other shrimp species, *F*. *aztecus* exhibits variations in its reproductive strategy, allocating reproductive effort temporally differently between latitudes [11].

The general latitudinal pattern observed in shrimp reproduction strategies is often referred to as ‘the classical paradigm’, and indicates continuous reproduction at low latitudes, with increased seasonality towards the poles [10,12–15]. We hypothesized that this latitudinal pattern reflects changes in the optimal reproductive strategy for different climate regimes along a latitudinal gradient, and investigated this hypothesis using a theoretical modeling approach. We used a stage-based density dependent matrix population model tailored to the life history of brown shrimp, with reproduction taking place either continuous, once, or twice a year. We modeled the effect of latitude by varying the length of the growing season and incorporating season-dependent parameters for carrying capacity and growth rate. Then, we investigated which of the three reproductive strategies resulted in the largest population size.

## Methods

### Matrix model

**Fig 1:**
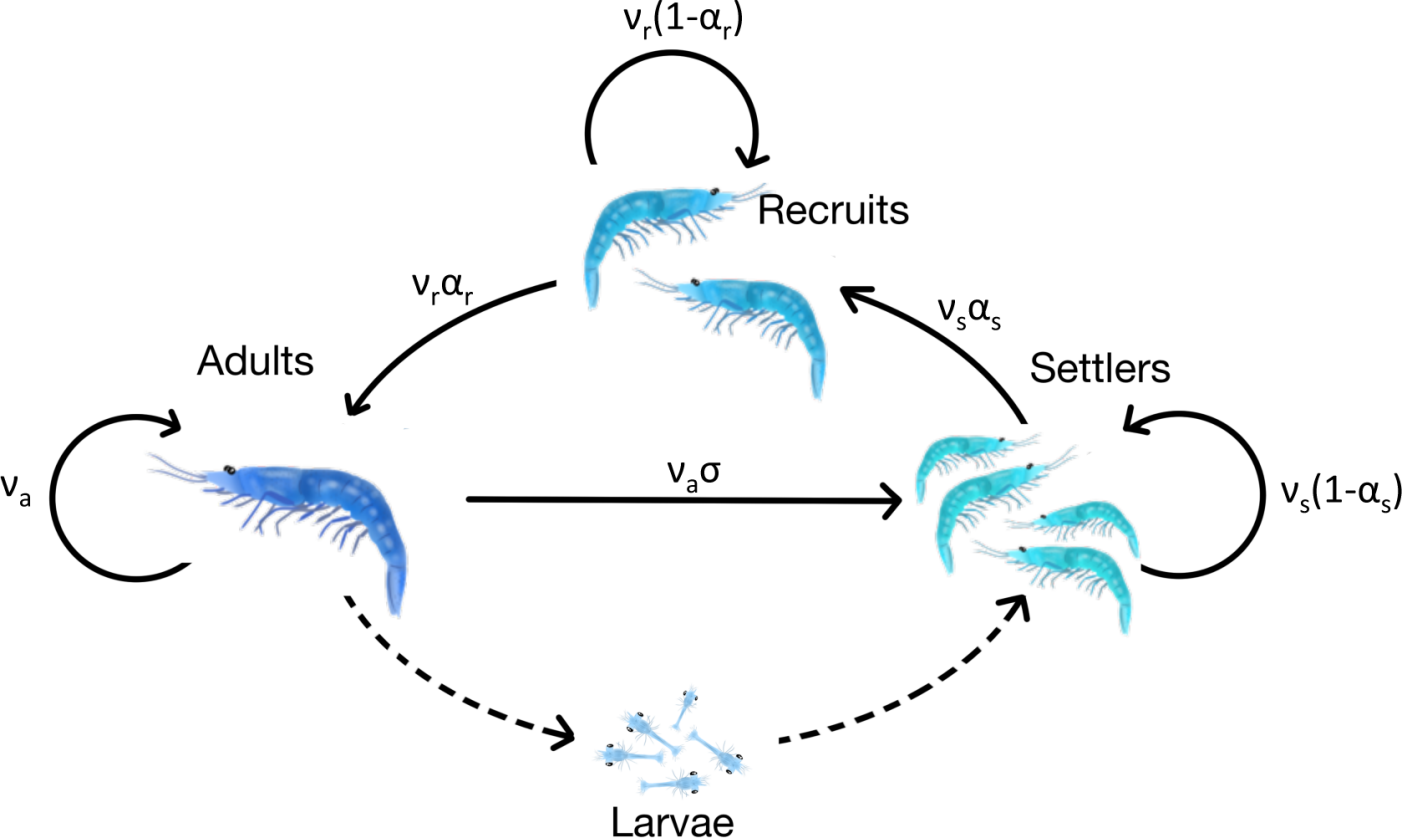
Life cycle diagram of the brown shrimp. Dotted arrows represent phases of the life history that were not included in the model. Solid lines indicate modeled stage transitions. *ν*_*i*_ represent survival rates, *α*_*i*_ the stage transition rates, and *σ* fertility.

We used a deterministic matrix model that simplified the brown shrimp life cycle into three primary stages, settler (s), recruit (r), and adult (a). Although shrimp do have a pelagic larval phase, there was insufficient data available to explicitly include a larval stage in the model. Therefore, we did not model this life phase explicitly in our model, but instead modeled reproduction by adding individuals directly to the settler stage (Fig 1). Thus, the settler stage represents juvenile shrimp that have survived the pelagic larval phase and have settled into estuaries, where they live until reaching approximately 75 mm in length, after which time they would be considered recruits and start migrating to open water bays and nearshore coastal areas [16–18]. Upon reaching 140 mm in length, brown shrimp were considered adults, sexually mature individuals typically found further offshore and targeted by larger commercial fishing operations.

The basic model can be described using conventional notation [19] as the following projection matrix:

$$A=\;\left[\begin{array}{ccc} \nu_{s}\left(1-\alpha_{s}\right) & 0 & \nu_{a}\sigma \\ \nu_{s}\alpha_{s} & \nu_{r}\left(1-\alpha_{r}\right) & 0\\ 0 & \nu_{r}\alpha_{r} & \nu_{a} \end{array}\right],$$

where ν_i_ represents the survival rate for each life stage, α_i_ is the fraction of individuals that transition to the subsequent stage, and σ is fertility, defined as the maximum number of individuals each adult spawner would add to the settler stage through reproduction, assumed constant. This fertility estimate was ultimately derived from the ratio of maximum recruits per adult spawner to estimated baseline settler survival, from literature [20]. Since brown shrimp have a fairly short lifespan and we were interested in the temporal allocation of reproduction efforts within the yearly time interval, we used a monthly time step.

### Parameter estimation

The growth rates, *γ*_*i*_, were assumed to be constant within each stage and based on field studies [21]. We divided growth rates by the stage size ranges, i.e. difference between the maximum (*L*_*i*,*max*_) and minimum (*L*_*i*,*min*_) length in stage *i*, to calculate transition rates, *α*_*i*_, for the settler and recruit stage classes (Eq 1).

$$\alpha_{i}=\frac{\gamma_{i}}{L_{i,max}-L_{i,min}}$$ (1)

Mortality (*M*_*i*_) in each stage *i* was calculated assuming a constant baseline instantaneous mortality rate multiplied by a size-dependent modifier:

$$M_{s}=0.6\;*\;53.092\;*\;L^{-1.1163}$$ (2)

to incorporate the inverse relationship between mortality and shrimp length, *L* [20,22]. To account for the demonstrated relationship between juvenile shrimp survival and the availability of vegetated marsh habitat, shrimp in the settler stage were subject to density-dependent survival [20,22–25]. We assumed a Beverton-Holt type of density dependence and calculated the settler survival rate (*ν*_*s*_) as

$$v_{s}\;=\;\frac{a_{1}}{1+b_{1}S}$$ (3)

where *a*_1_ is the maximum settler survival rate calculated as *1-M*_*s*_, with *S* denoting the settler abundance and *b*_1_ degree of density-dependence. To estimate the baseline degree of density dependence, we used semi-annual, fishery-independent abundance estimates collected by the Southeast Monitoring and Assessment Program (SEAMAP). We ran an initial simulation with survival, reproduction, and growth parameters fixed, based on literature as described above, then used a bounded constrained optimization alogorithm to estimate *b*_*1*_ and stage-specific catchability coefficients, *q*_*i*_.The latter terms were needed for direct comparison between model-predicted abundances and SEAMAP CPUE estimates. We ultimately selected the b1 that maximized fit between these values. Monthly reproduction (*σ*), i.e. the maximum rate at which new individuals were added to the settler stage class as a direct result of adult reproduction, was based on available literature [26]. All parameters values are summarized in Table 1.

**Table 1:** Parameter values used in the model.

Symbol	Description	Value
γ_ss_	Settler summer growth rate	30 mm
γ_sw_	Settler winter growth rate	5 mm
a_1_	Maximum settler survival rate	0.65
b_1s_	Summer degree of density dependence	8.34 E-9
b_1w_	Winter degree of density dependence	8.34 E-11
γ_r_	Recruit growth rate	13.07 mm
ν_r_	Recruit survival rate	0.69
ν_a_	Adult survival rate	0.73
σ	Monthly reproduction rate	7.70

### Reproductive strategies

The so-called ‘classical paradigm’ refers to continuous reproduction at low latitudes, and increasing seasonality towards the poles. To determine which reproductive strategy resulted in the largest population size and whether this varies with latitude, we modeled three different reproductive strategies. We made *σ* dependent on the month of the year *m*, i.e. *σ*_*m*_. To be able to assess potential differences between strategies, we assumed that all individuals had the same finite amount of resources to allocate towards reproduction. Therefore, the total annual output per individual (σ*12) was kept constant for all strategies so that only the temporal allocation of reproductive effort was altered. With the birth-pulse strategy, all reproductive effort is put into one high output reproductive event in March (*σ*_*March*_ = σ*12), with no reproduction (*σ*_*m*_ = 0) occurring in any other month. According to the paradigm, this strategy would be more favorable at higher latitudes. With the continuous strategy, the reproductive efforts are spread equally over the year. In our discrete model, this is represented by low output reproduction at every monthly time-step (*σ*_*m*_ = *σ*). The paradigm predicts that this type of reproduction is more favorable at low latitudes. Finally, since some shrimp populations have been observed to reproduce biannually, we incorporated this strategy by having reproduction in March (*σ*_*March*_ = σ*6) and September (*σ*_*September*_ = σ*6), but not in any other month (*σ*_*m*_ = 0).

### Latitudinal gradient

Older shrimp live at a water depth where the temperature does not change much depending on seasons, however; younger shrimp live at shallower depths and are affected by seasonality. Therefore, we modeled the effect of season on the settler stage. For simplicity, we only included two seasons in the model; a favorable season or ‘growing season’, and an unfavorable season. The effects of the different seasons on the population dynamics were incorporated into the model in two ways: (1) settlers generally grow faster in summer when the water temperatures and food availability are higher [22,27–29]. We modeled the average growth rate and consequently the transition rate of settlers to be higher in the favorable season, and (2) summer precipitation results in higher water levels and more habitat availability for settlers [30]. Thus, we modeled the carrying capacity of the environment for settlers to be higher in the favorable season by adjusting the b_1_ parameter (Table 1).

The latitudinal gradient was incorporated into the model as a change in the length of the growing season, assuming the climate is favorable all year in the tropics, but restricted to a few months per year at higher latitudes. To cover the complete range of possibilities, we let the length of the growing season range from 0 months (unfavorable conditions all year) to 12 months (favorable conditions all year) in steps of 1 month, resulting in 13 scenarios. For each scenario, we ran the model under the three reproductive strategies until equilibrium was reached. Population size was highly temporally variable due to the birth peaks and density dependence. Thus, we calculated the average number of adults through an annual cycle. Comparisons between scenarios and reproductive strategies were based on these average adult population sizes at equilibrium. Since we looked at equilibrium values, the results were not affected by the population sizes used to initialize the model.

## Results

**Fig 2:**
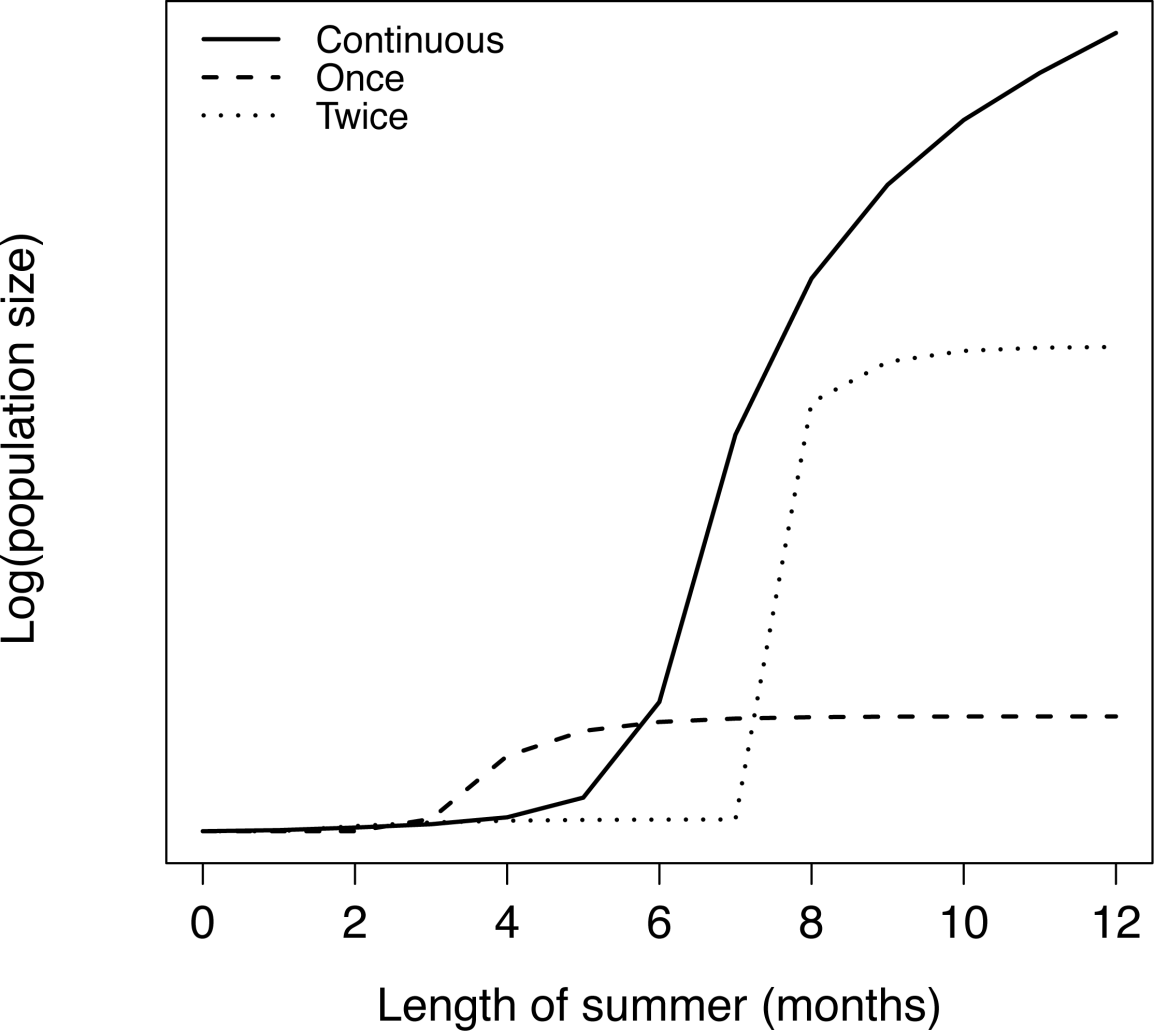
Average annual adult population size for each of the three reproductive strategies for different summer lengths.

Growing season duration had the most dramatic effect on the average adult population size under all reproductive strategies. Shrimp populations were not viable when the growing season was less than 3 months. As the growing season became longer, the singe birth-pulse strategy resulted in the largest adult population size, until the growing season reached 6 months (Fig 2). When growing season exceeded 6 months, the continuous reproduction strategy was most prolific, resulting in increasingly larger population sizes than the other. Although the double birth-pulse strategy resulted in larger population sizes than the single-pulse strategy for growing seasons longer than about 7 months, it was never the most optimal strategy for reproductive purposes.

**Fig 3:**
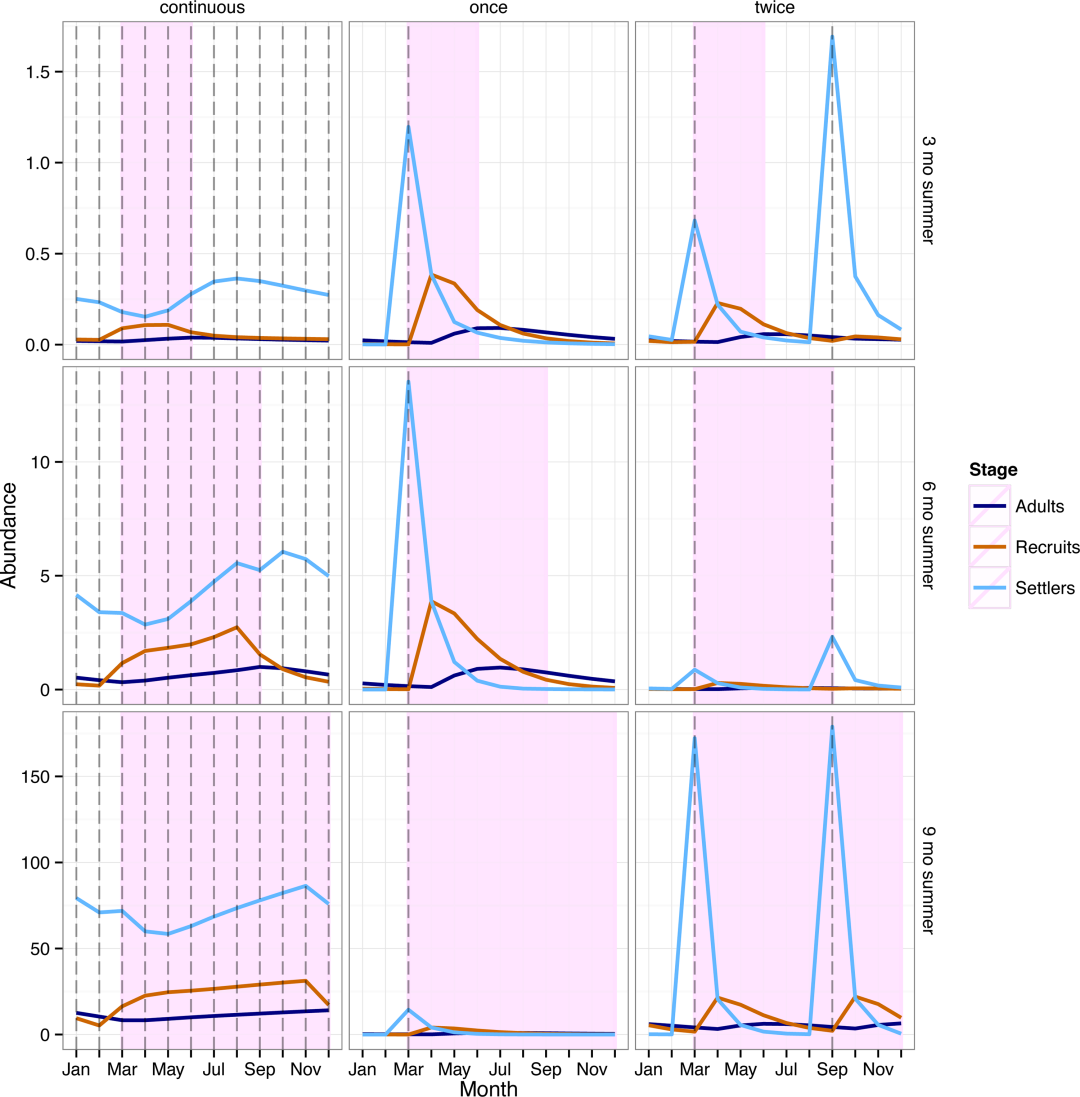
Number of individuals in each stage at equilibrium through one annual cycle for each reproductive strategy and summer lengths of 3, 6 and 9 months. The vertical dashed lines indicate reproductive events. Shaded area indicates summer. Note the different scales on the y axes.

The underlying population dynamics were strongly influenced by the density dependent survival of the settlers. There were typically large differences in stage-specific abundances because only a small fraction of individuals in the settler stage transitioned to the recruit and adult stages (Fig 3).

## Discussion

Species adapt to climatic variations along latitudinal gradients by adjusting their reproductive strategy. We investigated how the temporal allocation of reproductive effort in brown shrimp affected their equilibrium adult population size. We demonstrated that continuous reproduction resulted in larger population sizes at low latitudes, while reproducing in one birth-pulse lead to a larger population size at higher latitudes. These results are consistent with the classical paradigm of continuous reproduction around the equator, and increasing seasonality in reproduction towards the poles.

When investigating life history traits, researches typically optimize measures of fitness such as lifetime reproductive success or population growth rate. Since we examined populations at their equilibrium, when the growth rate is always 0 and every individual only replaces itself, these common measures did not lend themselves for comparison. Nonetheless, higher reproduction rates and faster population growth in our model did lead to larger populations, thus, we used population size as a proxy for the more traditional measures. Additionally, population size is highly correlated with population persistence [31], which is of interest in fisheries studies.

To successfully parse out the effects of temporal allocation of reproductive effort on population size, we made several simplifying assumptions that may limit the direct extrapolation of our results to field applications. We first assumed that the total annual reproductive output of adult shrimp was constant. Since resources are limited, there are trade-offs between the amount of energy spent on different life history components [32]. Without specific data on brown shrimp energy allocation, we assumed that the total amount of energy spent on reproduction in one year was the same for all strategies and all latitudes. We also did not account for potential differences in food availability that could be correlated with latitudinal variations, and potentially drive the selection of one reproductive strategy over another. An investigation of wild-caught adult brown shrimp reared in captivity did find varied reproductive output related to diet quality [33]. However, differences in potential diet quality and quantity available to adult brown shrimp at various latitudes was well beyond the scope of this study. Notwithstanding the unknown implications of diet, the somewhat simplistic assumptions regarding climate did not hinder our ability to compare reproductive strategies because the unknown latitudinal variation would likely be similar for each of them.

Another assumption in our models was that the latitudinal variation in climate is accurately represented by changes in the length of summer. Juvenile shrimp have been shown to grow faster or slower depending on the temperature [22,27–29]. At low latitudes, the temperature is favorable year round, but as the distance from the equator increases, the temperature is unfavorable for progressively longer periods of time. Therefore, the ‘favorable season’ is year round in the tropics, and decreasing in length towards the poles. Within the favorable temperature range, juvenile shrimp grow faster at high temperatures and slower at lower temperatures [27]. We did not incorporate this level of detail in our modeling, but rather choose an average growth rate for the favorable season. Since the temperatures in the favorable season at low latitudes are higher than those at higher latitudes, juvenile shrimp grow faster in the tropics [34]. Thus, the population differences between our birth-pulse and continuous reproduction strategies in a field setting might be larger than model predictions.

Finally, we assumed that carrying capacity and growth rate in the settler stage were the primary underlying demographic processes affected by seasonality. While seasonal changes in temperature, water level and salinity are not as significant for larger shrimp in deeper water, they can drastically affect the shallow habitat of settlers, and settler abundance may be the most significant predictor of abundance in later stages [18,29,35]. Therefore, our model focused exclusively on settlers when incorporating potential seasonal effects. Besides temperature, water level was considered another major component of the carrying capacity. In the Gulf of Mexico, moderately high water levels increase the amount of habitat available to settlers [22,30,36,37]. In our model, we incorporated these potential benefits by increasing the carrying capacity for settlers during the favorable season.

Simulation results demonstrated the potential effects of alternate reproductive strategies on adult shrimp abundance. This type of information is critical when considering optimal harvest and management policies for commercial shrimp species at varying latitudes. In the brown shrimp fishery, simulation models using catch information from Texas have included several factors influencing optimal harvesting patterns, *i*.*e*. fishing effort, market price, shrimp supply from other states or imports, fuel costs, and recruitment [38–41]. In general, the fishery has been managed to prevent overfishing by setting catch-limits on smaller sub-adult shrimp and closing the inland (*i*.*e*. within 9 nmi of the shoreline) fishery altogether during key periods to allow shrimp to obtain larger sizes and migrate offshore [40–43]. These management strategies are based on reported life history patterns of shrimp within the general latitudinal range of the northern Gulf of Mexico, which include a primary period of brown shrimp larvae recruitment into marsh areas between February and April, and emigration of post-larvae into coastal Bays and offshore areas from May through July [16,17,42,44].

Observed brown shrimp dynamics in the Gulf of Mexico most closely resemble the single birth pulse strategy (Fig 2). If brown shrimp have evolved to exploit the most effective reproductive strategy, environmental conditions in the Gulf seem to support an annual ‘growing season’ of 6 months or less. Conversely, a recent study of a Caribbean penaeid shrimp species, *Farfantepenaeus notialis*, noted a bi-annual reproductive strategy with birth peaks in Apr-June, then again Oct-Dec [45]. This observation is consistent with our model, which predicts that a double birth-pulse is more advantageous than a single birth-pulse given a longer (>7 months) growing season. If we assume that temperature is one of the dominant covariates of ‘growing season’, the lower latitude and milder Caribbean climate could explain why *F*. *notialis* has evolved to exploit two reproductive peaks, while a single birth-pulse is found in Gulf of Mexico *F*. *aztecus*. Resource agencies are still in the early stages of developing optimal management strategies for the former species, and one could reasonably assume any proposed fishery closures would need to take the dual birth peaks into consideration. While our model results suggested that a continuous reproduction strategy would be optimal when the growing season exceeded six months, the assumption that reproductive output would be evenly distributed over that entire period was most likely over-simplified. Pérez-Castañeda and Defeo [35] conducted a field study of four *Farfantepenaeus* species in the Yucatan Peninsula over multiple seasons and found that recruits (shrimp < 8 mm) were found throughout the year, suggesting continuous reproduction, but there were also two clear reproductive peaks, *i*.*e*. a double birth-pulse strategy. While the results are somewhat confounded by an inability to identify species-specific recruit abundance, there was still strong evidence suggesting reproduction that is indeed continuous, but with greater output in two key periods. In fact, this hybrid strategy seems reasonable given an overall mild sub-tropical climate that favors some year-round fitness, but still with climatic seasons (*i*.*e*. dry, rainy and nortes) that affect the magnitude of the ‘growing season’ [35].

Understanding the role varying latitudes play in the reproductive strategies of commercially important fisheries species like brown shrimp can be crucial to developing optimal management strategies. In the case of *P*. *aztectus* in the Gulf of Mexico, strategies that include closing the fishery during key periods of post-larvae emigration are well aligned with the observed single-birth pulse event. However, climate variability could alter the timing and duration of the growing season. For example, an extended growing season brought on by warming temperatures could shift dynamics toward more continuous reproduction. Unfavorable environmental conditions, *i*.*e*. prolonged hypoxia [46], that shorten the growing season below the 3 month threshold, could crash susceptible areas of the fishery altogether. Our model predicted a shift in the optimal strategy, from one annual birth pulse with high reproductive output, to continuous low-output reproduction as the growing season duration increased toward lower latitudes. Such a result is consistent with the classical paradigm, which indicates that continuous reproduction at low latitudes produces the greatest population abundance. Future studies looking at how projected climate change might affect the timing and periodicity of reproduction, especially for commercially important fisheries species such as brown shrimp, are certainly warranted to facilitate optimal management and harvest of these species.

## References

[1] Giesel JT. Reproductive strategies as adaptations to life in temporally heterogeneous environments. Annu Rev Ecol Syst. 1976;7(1):57–79.

[2] Bye V. The role of environmental factors in the timing of reproductive cycles In: Potts G, Wootton R, editors. Fish reproduction: strategies and tactics. Academic Press, London, United Kingdom; 1984 p. 187–205.

[3] Winemiller K. Patterns of variation in life-history among south-american fishes in seasonal environments. Oecologia. 1989;81(2):225–41. 10.1007/BF00379810 28312542

[4] Thorson G. Reproductive and larval ecology of marine bottom invertebrates. Biol Rev. 1950;25(1):1–45. 24537188 10.1111/j.1469-185x.1950.tb00585.x

[5] Colihueque N, Cárdenas R, Ramírez L, Estay F, Araneda C. Analysis of the association between spawning time QTL markers and the biannual spawning behavior in rainbow trout *(Oncorhynchus mykiss)*. Genet Mol Biol. 2010;33(3):578–82. doi: 10.1590/S1415-47572010000300032 21637435 10.1590/S1415-47572010000300032 PMC3036128

[6] Kawamichi T. Biannual reproductive cycles in the Japanese giant flying squirrel *(Petaurista leucogenys)*. J Mammal. 2010;91(4):905–13.

[7] Fransozo A, Leão Castilho A, Simões SM, D’Incao F, Caetano da Costa R. Sex ratio, growth and recruitment of the pelagic shrimp *Acetes americanus* on the southeastern coast of Brazil. J Crustac Biol. 2013;33(1):1–9.

[8] Elliot JA, Goldman BD. Seasonal reproduction In: Adler NT, editor. Neuroendocrinology of Reproduction. Springer, Boston, MA, USA.; 1981 p. 377–423.

[9] Nichols JD, Conley W, Batt B, Tipton AR. Temporally dynamic reproductive strategies and the concept of R- and K-selection. Am Nat. 1976;110:976–95.

[10] Leggett W, Carscadden J. Latitudinal variation in reproductive characteristics of american shad *(Alosa sapidissima)*—evidence for population specific life-history strategies in fish. J Fish Res Board Canada. 1978;35(11):1469–78.

[11] Bauer RT. Testing generalizations about latitudinal variation in reproduction and recruitment patterns with sicyoniid and caridean shrimp species. Invertebr Reprod Dev. 1992;22(1–3):193–202.

[12] da Costa RC, Branco JO, Machado IF, Campos BR, Avila MG. Population biology of shrimp *Artemesia longinaris* (Crustacea: Decapoda: Penaeidae) from the southern coast of Brazil. J Mar Biol Assoc United Kingdom. 2010;90(4):663–9.

[13] Aragon-Noriega EA. Coupling the reproductive period of blue shrimp *Litopenaeus stylirostris* (Stimpson, 1874, Decapoda: Penaeidae) and sea surface temperature in the Gulf of California. 2007;42(2):167–75.

[14] Fransozo A, da Costa RC. Reproductive biology of the shrimp *Rimapenaeus constrictus* (Decapoda, Penaeidae) in the Ubatuba region of Brazil. J Crustac Biol. 2004;24(2):274–81.

[15] Castilho AL, Costa RC, Fransozo A, Boschi EE. Reproductive pattern of the South American endemic shrimp Artemesia longinaris (Decapoda: Penaeoidea), off Sao Paulo State, Brazil. Rev Biol Trop. 2007;55:39–48.

[16] Williams AB. A contribution to the life histories of commercial shrimps (Penaeidae) in North Carolina. Bull Mar Sci Gulf Caribb. 1955;5:116–59.

[17] Knudsen E, Paille R, Rogers B, Herke W, Geaghan J. Effects of a fixed-crest weir on brown shrimp *Penaeus aztecus* growth, mortality, and emigration in a Louisiana coastal marsh. North Am J Fish Manag. 1989;9:411–9.

[18] Haas HL, Lamon EC III, Rose KA, Shaw RF. Environmental and biological factors associated with the stage-specific abundance of brown shrimp *(Penaeus aztecus)* in Louisiana: applying a new combination of statistical techniques to long-term monitoring data. Can J Fish Aquat Sci. 2001;58(11):2258–70.

[19] Caswell H. Matrix Population Models: Construction, Analysis, and Interpretation. 2nd ed. Sunderland: Sinauer Associates, Inc; 2001.

[20] Haas HL, Rose KA, Fry B, Minello TJ, Rozas LP. Brown shrimp on the edge: linking habitat to survival using an individual-based simulation model. Ecol Appl. 2004;14(4):1232–47.

[21] Rose CD, Harris AH, Wilson B. Extensive Culture of Penaeid Shrimp in Louisiana Salt-marsh Impoundments. Trans Am Fish Soc. Taylor & Francis Group; 1975 4 9;104(2):296–307.

[22] Minello T, Zimmerman R, Martinez E. Mortality of young brown shrimp *Pinaeus aztecus* in estuarine nurseries. Trans Am Fish Soc. 1989;118(6):693–708.

[23] Minello T, Zimmerman R. Fish predation on juvenile brown shrimp, *Penaeus aztecus*—effects of simulated Spartina structure on predation rates. J Exp Biol Ecol. 1983;72:211–31.

[24] Childers D, Day J, Muller R. Relating climatological forcing to coastal water levels in Louisiana estuaries and the potential importance of El Nino—Southern oscillation events. Clim Res. 1990;1:31–42.

[25] Minello TJ, Rozas LP, Baker R. Geographic variability in salt marsh flooding patterns may affect nursery value for fishery species. Estuaries and Coasts. 2011;35(2):501–14.

[26] Beukema J. Dynamics of juvenile shrimp *Crangon crangon* in a tidal-flat nursery of the Wadden Sea after mild and cold winters. Mar Ecol Prog Ser. 1992;83(2–3):157–65.

[27] Zein-Eldin ZP, Aldrich D V. Growth and survival of postlarval Penaeus aztecus under controlled conditions of temperature and salinity. Biol Bull. 1965;129(1):199.

[28] Zein-Elding Z, Renaud M. Inshore environmental effects on brown shrimp, *Penaeus aztecus*, and white shrimp, *P*. *setiferus*, populations in coastal waters, particularly of Texas. U S Natl Mar Fish Serv Mar Fish Rev. 1986;48:9–19.

[29] Li J, Clarke AJ. Sea surface temperature and the brown shrimp *(Farfantepenaeus aztecus)* population on the Alabama, Mississippi, Louisiana and Texas continental shelves. Estuar Coast Shelf Sci. 2005;64(2–3):261–6.

[30] Minello TJ, Rozas LP, Baker R. Geographic variability in salt marsh flooding patterns may affect nursery value for fishery species. Estuaries and Coasts. 2012;35(2):501–14.

[31] Reed DH. Relationship between population size and fitness. Conserv Biol. 2005;19(2):563–8.

[32] Fisher RA. The genetical theory of natural selection Oxford Clarendon Press; 1930. 306 p.

[33] Gandy, Ryan L. Investigations into the reproductive performance and larval rearing of brown shrimp, *Farfantepenaeus aztecus* using closed recirculating systems Texas A&M University; 2004.

[34] Baker R, Fujiwara M, Minello TJ. Juvenile growth and mortality effects on white shrimp *Litopenaeus setiferus* population dynamics in the northern Gulf of Mexico. Fish Res. 2014;155:74–82.

[35] Pérez-Castañeda R, Defeo O. Population variability of four sympatric Penaeid shrimps *(Farfantepenaeus spp*.*)* in a tropical coastal lagoon of Mexico. Estuar Coast Shelf Sci. 2001;52(5):631–41.

[36] Minello T, Zimmerman R. Utilization of natural and transplanted Texas salt marshes by fish and decapod crustaceans. Mar Ecol Prog Ser. 1992;90(3):273–85.

[37] Minello T, Rozas L. Nekton in Gulf Coast wetlands: Fine-scale distributions, landscape patterns, and restoration implications. Ecol Appl. 2002;12(2):441–55.

[38] Grant W, Griffin W. Bioeconomic model of the gulf of Mexico shrimp fishery. Trans Am Fish Soc. 1979;108(1):1–13.

[39] Onal H, McCarl BA, Griffin WL, Matlock G, Clark J. A bioeconomic analysis of the Texas shrimp fishery and its optimal management. Am J Agric Econ. 1991;73(4):1161–70.

[40] Cole JG, Gallaway BJ, Martin LR, Nance JM, Longnecker M. Spatial allocation of shrimp catch based on fishing effort: adjusting for the effects of the Texas opening. North Am J Fish Manag. 2006;26(4):789–92.

[41] Caillouet CW, Hart RA, Nance JM. Growth overfishing in the brown shrimp fishery of Texas, Louisiana, and adjoining Gulf of Mexico EEZ. Fish Res. 2008;92(2–3):289–302.

[42] Nance J, Martinez E, Klima E. Feasibility of improving the economic return from the Gulf of Mexico brown shrimp fishery. North Am J Fish Manag. 1994;14:522–36.

[43] Ward JM, Sutinen JG. Vessel entry-exit behavior in the Gulf of Mexico shrimp fishery. Am J Agric Econ. 1994;76(4):916.

[44] Zimmerman RJ, Minello TJ. Densities of *Penaeus aztecus*, *Penaeus setiferus*, and other natant macrofauna in a Texas salt marsh. Estuaries. 1984;7(4):421.

[45] Paramo J, Perez D, Wolff M. Reproduction of the pink shrimp *Falfantepenaeus notialis* (Decapoda: Penaeidae) in the Colombian Caribbean. Rev Biol Trop. 2014;62(2):513–21. 25102635

[46] Huang L, Smith MD. Management of an annual fishery in the presence of ecological stress: The case of shrimp and hypoxia. Ecol Econ. 2011;70(4):688–97.

